# Vaccination against *Lawsonia intracellularis* reduced tail-biting related behaviors in a commercial pig herd

**DOI:** 10.1186/s40813-026-00530-4

**Published:** 2026-07-16

**Authors:** Déborah Temple, Ivan Díaz, Eva Mainau, Rosa Centelles, Damián Escribano, César Llorente, Llorenç Badiella, Stephan von Berg, Marta Jiménez

**Affiliations:** 1 AWEC, Edifici Eureka, Campus de la Universitat Autònoma de Barcelona, Barcelona, Spain; 2 Department Sanitat i d’Anatomia Animals, Facultat de Veterinària, Universitat Autònoma de Barcelona, Barcelona, Spain; 3 La Manresana, Els Prats de Rei, Spain; 4 Salilab, UMU Interdisciplinary Laboratory of Clinical Analysis, Faculty of Veterinary Science, University of Murcia, Murcia, Spain; 5 Department of Animal Production, University of Murcia, Murcia, Spain; 6 MSD Animal Health, Cargajosa de la Sagrada, Spain; 7 Department de Matemàtiques, Universitat Autònoma de Barcelona, Barcelona, Spain; 8 MSD Animal Health, Munich, Germany

**Keywords:** *Lawsonia intracellularis*, Pigs, Behavior, Tail biting, Welfare, Vaccination, Gut health, Biomarkers

## Abstract

**Background:**

*Lawsonia intracellularis* is the causative agent of proliferative enteropathy, a common enteric disease in pigs that compromises intestinal integrity and may influence behavior through inflammation and immune activation. Its potential link to tail biting, an abnormal behavior that causes stress, injury and pain, remains unexplored. This study evaluated the effect of vaccination against *L. intracellularis* on tail-biting related behaviors, tail lesions, active behaviors, and postures in pigs reared under commercial conditions.

**Methods:**

The study compared two groups in a commercial herd naturally and subclinically infected with *L. intracellularis*: pigs vaccinated against *L. intracellularis* at 5 weeks of age and unvaccinated controls each comprising 21 pens observed from 11 to 28 weeks of age. Pigs had intact tails. *L. intracellularis* DNA in feces and specific antibodies in blood were detected by qPCR and ELISA, respectively. Tail-biting related behaviors (rear-end nosing and tail manipulation) were quantified by video analysis (*n* = 42 pens). Tail lesions were assessed weekly and individually for each pig (*n* = 516 pigs) using a standardized scoring system. Active behaviors (exploration, feeding, social interactions) and postures were recorded as additional behavioral measures. Haptoglobin (Hp), C-reactive protein (CRP), total esterase activity (TEA) and adenosine deaminase (ADA) were analyzed in blood or saliva. Mortality and average daily gain (ADG) were registered. Ethical welfare management measures (temporary space increase, tail spraying, and enrichment) were applied when recurrent or repeated bleeding from the tail was observed.

**Results:**

*L. intracellularis* was detected between 15 weeks of age and 24 weeks of age. During this period, vaccinated pigs exhibited lower frequency of tail-biting related behaviors, whereas tail lesion scores did not differ between groups. Unvaccinated control pigs explored enrichment more frequently and were sporadically more active and restless while vaccinated pigs spent more time at the feeder. ADA concentrations were higher in vaccinated pigs, while Hp, CRP, TEA, mortality, and ADG did not differ (*p* > 0.05).

**Conclusions:**

Under natural subclinical *Lawsonia intracellularis* infection, vaccinated pigs demonstrated fewer tail-biting related behaviors and lower levels of active behaviors compared with unvaccinated controls. Although tail lesion rates were similar between groups, likely due to effective welfare management measures, the observed behavioral differences suggest that vaccination against *L. intracellularis* can enhance pig welfare even in the absence of clinical disease. Therefore, controlling subclinical enteric infections through vaccination may serve as an indirect yet practical complementary strategy to reduce tail-biting related behaviors on commercial pig farms.

**Supplementary Information:**

The online version contains supplementary material available at https://doi.org/10.1186/s40813-026-00530-4.

## Background

*Lawsonia intracellularis* is an obligate intracellular bacterium that causes proliferative enteropathy, a common enteric disease in growing and finishing pigs. The infection is characterized by hyperplasia of intestinal crypts, mucosal thickening, and inflammation. Clinical outcomes range from diarrhea and reduced feed efficiency to increased morbidity and mortality in severe cases [1, 2]. Beyond its impact on performance, *L. intracellularis* infection compromises gut integrity, including epithelial structure and barrier function, and may induce discomfort and behavioral changes associated with local inflammation and systemic immune activation [3, 4]. Vaccination against *L. intracellularis* has proven effective in reducing bacterial shedding, mortality, and clinical signs under both experimental and field conditions [5–9]. However, the potential behavioral and welfare benefits of vaccination, stemming from improved gastrointestinal (GI) health, have not been fully elucidated.

The GI tract communicates with the nervous and immune systems through bidirectional neuroimmune signaling, known as the gut-brain axis. Perturbations in this system, driven by inflammation or microbiota shifts [10], can therefore alter motivation and normal behavior. In pigs, some studies have demonstrated that health challenges can significantly impact behavior and activity patterns; specifically, animals housed under poor sanitary conditions or exposed to suboptimal health conditions may exhibit an increase in damaging behaviors, including ear and tail biting [11–14]. Similarly, pigs suffering from digestive disturbances or discomfort have been linked to restlessness and increased exploration, suggesting that abdominal discomfort may trigger negative affective states [15].

Tail biting remains one of the most persistent welfare challenges in pig production, causing stress, pain, infection risk, and significant economic losses [16, 17]. Tail biting is a multifactorial behavior, with extensive literature identifying a wide range of contributing factors, risk conditions, and mitigation strategies [18]. A widely accepted view is that tail biting represents an abnormal behavior expressed under barren housing conditions, arising from limitations on pigs’ ability to perform natural rooting, foraging, and exploratory behaviors [19]. Many stressful challenges, including environmental and management factors, social factors and individual factors, have been identified as risks for tail biting. While environmental and management risk factors have been widely investigated, the contribution of physiological or health-related drivers remains much less explored. Few studies have demonstrated a significant link between enteric health and tail-biting behavior [11]. Distinct gut microbiota compositions have been identified in biters, victims, and neutral pigs [20, 21], supporting a connection between intestinal health and tail biting behavior.

Given the widespread prevalence of *L. intracellularis* and its impact on gut health, it is plausible that pigs experiencing enteric inflammation may exhibit altered activity and an increased risk of redirected oral manipulation such as tail biting behavior. Consequently, improving gut integrity through vaccination could help mitigate these welfare issues. However, this potential link between enteric health, vaccination against GI disorders, and pig behavior remains untested.

To complement behavioral observations, biomarkers can be used to capture physiological processes associated with subclinical enteric challenges. Infection with *Lawsonia intracellularis* is associated with intestinal inflammation and immune activation; accordingly, haptoglobin (Hp) and C-reactive protein (CRP) are commonly used indicators of systemic inflammatory responses in pigs [4, 22–24]. Total esterase activity (TEA) in saliva provides a non-invasive measure of autonomic activation related to stress or pain [25, 26]. In addition, adenosine deaminase (ADA) activity reflects cell-mediated immune activation, which is considered central to protective immune response against *Lawsonia intracellularis* [27, 28]. Together, these biomarkers provide physiological context for behavioral outcomes by distinguishing between systemic inflammation, stress-related responses, and immune activation.

This study compared vaccinated and unvaccinated pigs on a commercial farm with subclinical *Lawsonia intracellularis* infection to investigate whether vaccination influences tail-biting related behaviors, active behaviors, postures, and tail lesion severity. Behavioral observations were complemented by biomarkers of inflammation, acute stress, and immune activation.

## Materials and methods

### Animals, housing, and general management

The study was conducted on a commercial growing-finishing pig farm located in Spain. A total of 516 pigs (Duroc x Berkshire) with intact tails were included. Pigs were individually identified at the farrowing unit within the first 7 days of age using electronic RFID (radio-frequency identification) ear tags (LeeO^®^, MSD Animal Health). No specific selection criteria were applied, and all animals were managed in accordance with standard farm practices. Males were surgically castrated within the first 7 days of age under analgesia, using meloxicam (Metacam^®^), a non-steroidal anti-inflammatory drug. Pigs came from 83 sows and were mixed at weaning at an average age of 26 days (± SD 3.1). All animals spent 5 weeks at the weaning unit before being transported to the finishing building, approximately 30 min away, by a commercial trailer. After allocation to their finishing pens, no mixing was performed during the study period, except when animals were moved to hospital pens if required.

The pigs were housed in a single finishing building comprising 42 study pens, with an average of 12 pigs per pen. Pens were single sex (either males or females), and the entire building was managed under an all-*in*,* all-out* production system. Average stocking density was 0.9 m² per pig when entering the finishing unit at 10 weeks of age. Pens were equipped with fully slatted concrete floors (tread area 150 mm width) and solid partitions, which prevented physical and visual contact between animals in neighboring pens. Pigs had *ad libitum* access to commercial dry feed for growing-finishing pigs and to water provided via nipple drinkers integrated on both sides of the trough (Maximat^®^ Porker).

**Fig. 1:**
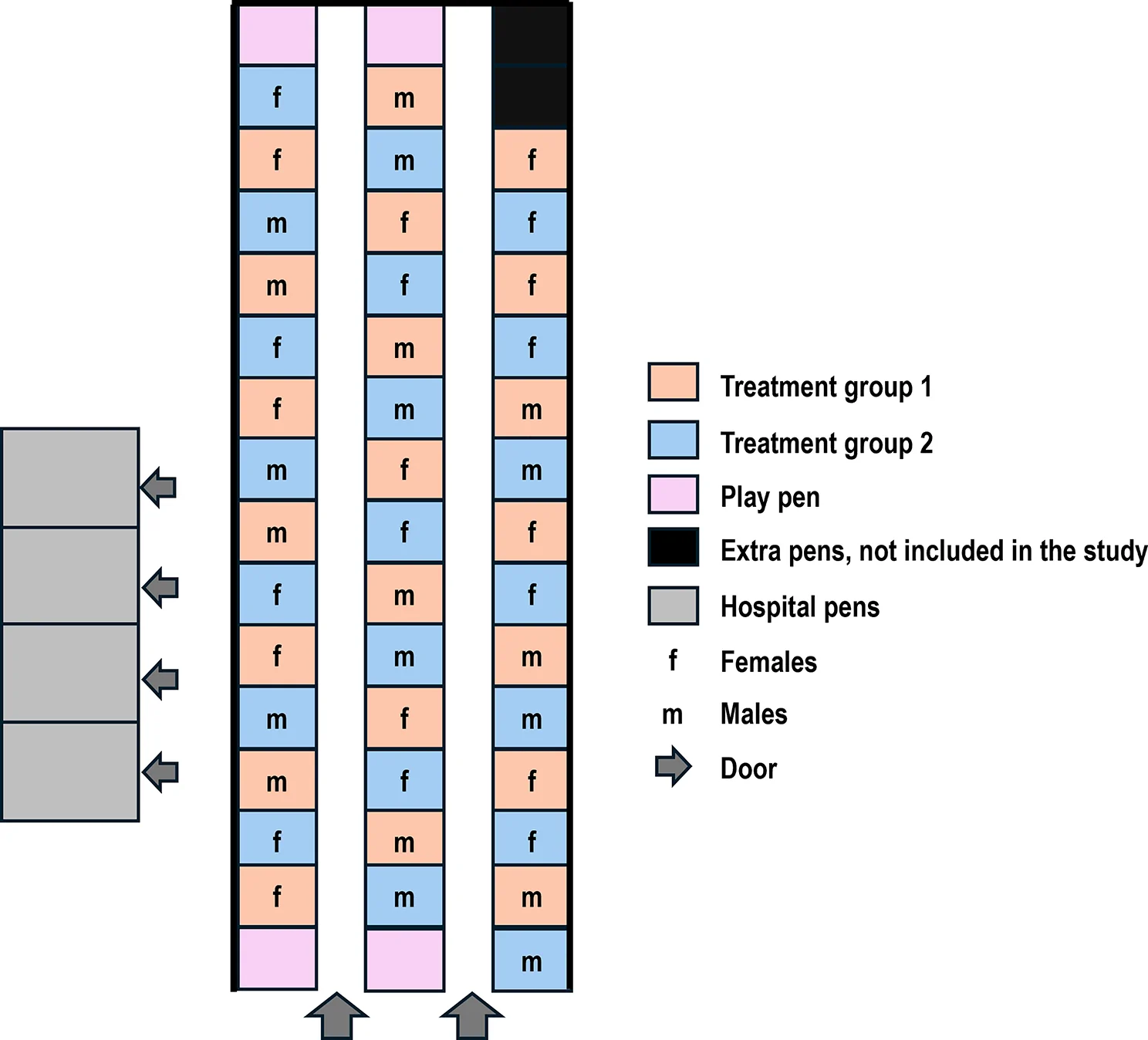
Schematic representation of the building layout, including pen types, treatment allocation, and sex distribution

**Fig. 2:**
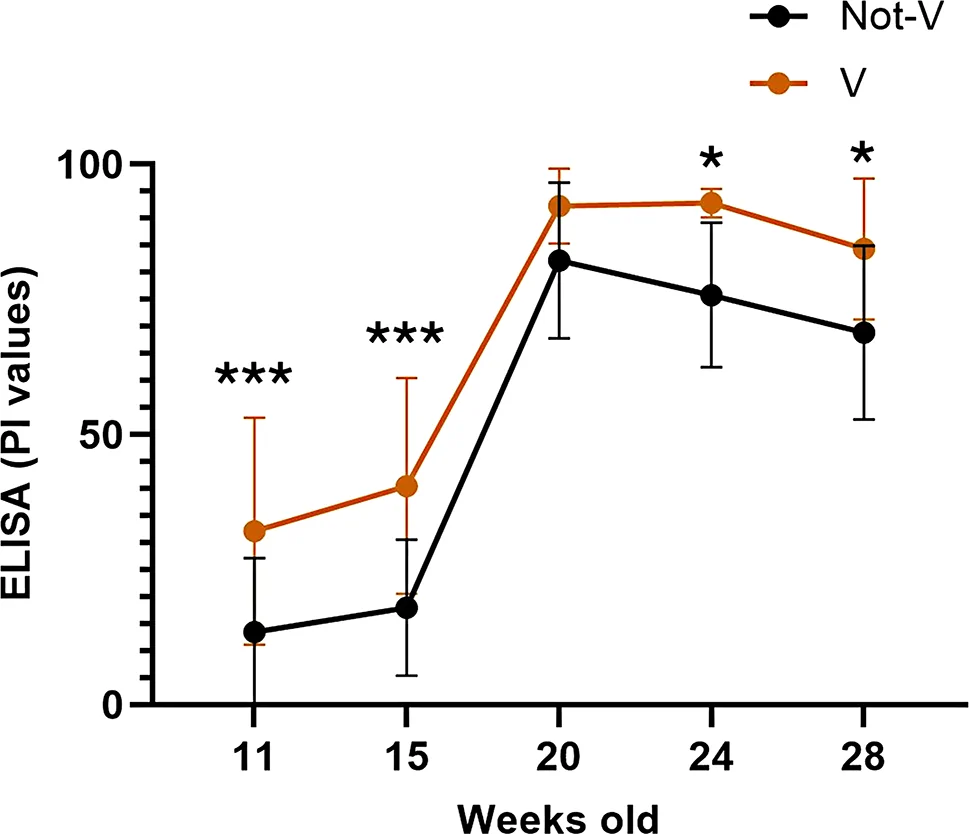
Evolution of specific antibodies against *L. intracellularis* (PI values) from 84 pigs. Significant differences between the two treatment groups (unvaccinated = Not-V and vaccinated = V) are shown. * *p* < 0.05, ** *p* < 0.01, *** *p* < 0.001

Management followed the farm’s standard practices. The building was naturally ventilated, and lighting was provided through natural daylight from windows, supplemented by artificial light in the morning (from 07:30) and in the evening (from 18:00). Environmental enrichment was provided in each pen using a rotational system of twelve different toys (e.g. EasyFix Astro 200^®^ rubber material; rod-shaped toys). Enrichment items were exchanged twice weekly (every Mondays and Fridays) throughout the finishing period to maintain novelty and interest. Four additional pens were used as “play pens” when tail-biting outbreaks were detected, and four other pens, located in an adjacent smaller building, were used as hospital pens. Play pens and hospital pens were not included in the study. During the trial, 20 pigs were treated with amoxicillin (Amoxal^®^) and 9 with benzylpenicillin (Depocillin^®^), administered at the individual level (13 pigs in the control group and 16 in the vaccinated group). Ambient temperature and relative humidity were monitored throughout the entire period (Additional File 1, AF Fig. 1 and AF Fig. 2).

### Experimental design

The finishing cycle under study extended from July to November 2024. A total of 516 pigs in the 42 pens were included in the study. At 5 weeks of age in the weaning unit, pens were randomly allocated to either the vaccination or control groups. Consequently, all pigs within a given pen were either intradermally vaccinated against *Lawsonia intracellularis* (0.2 mL Porcilis^®^ Lawsonia ID, MSD Animal Health) or left unvaccinated. At entry to the finishing unit, pigs - previously identified with RFID ear tags at the farrowing unit - were individually weighed and allocated to pens according to treatment group, sex, and body weight. Each finishing pen contained pigs from a single treatment group (vaccinated or unvaccinated) and a single sex, with efforts made to ensure homogeneous weight distribution. The allocation process aimed to balance treatment groups across the facility and avoid spatial clustering (Fig. 1). All other management practices were identical across treatment groups. Vaccination was administered at the weaning unit by farm staff and a trained technician and was therefore not blinded at the time of treatment. Treatment allocation was subsequently recorded in the LeeO^®^ management software for each individually identified pig and associated with a color code. All personnel involved in behavioral observations, tail lesion scoring, laboratory analyses, and statistical analyses were blinded to treatment allocation. Treatments were revealed only after completion of the statistical analyses.

An overview of the procedures conducted during the trial, the outcome measures collected, and the statistical models applied is provided in Table 1.

**Table 1:** Summary of procedures conducted during the trial, including outcome measures and statistical model applied

	Sampling, *N* and Observer	Timing and procedures	Outcome measures and statistical models
Behavioral observations – video recordings – focal sampling	Pen level Video recording *N* = 42 pens Observer Nº1	Weekly, every 7 days (Thursdays) Starting 7 days after arriving (11 weeks old) and until 28 weeks old	Nosing tail region Tail manipulation Ear manipulation Belly nosing Number of events per 10 pigs within 30 min Poisson offset – GLMM
Behavioral observations – video recordings - instantaneous scan sampling	Pen level Video recording *N* = 42 pens Observer Nº2	Weekly, every 7 days (Thursdays) Starting 7 days after arriving (11 weeks old) and until 28 weeks old	Active behaviors (see Table 3) Proportion to the number of active pigs Binomial - GLMM Postures (see Table 3) Proportion to the number of pigs in each posture in relation to the total number of pigs observed Binomial- GLMM
Tail lesion scoring – direct evaluation at the farm	Individual level *N* = 516 pigs Observer Nº3	Weekly, every 7 days (Fridays) Starting at arrival at the farm (10 weeks old) and until 29 weeks old	Four categories of tail lesions (see Table 4) Proportion of affected pigs for each tail lesion category in relation to the total number of pigs assessed Binomial- GLMM
Detection of *L. intracellularis* - serology	Individual level *N* = 84 pigs, two pigs per pen (42 pens) Blood samples were sent to the lab at the end of the study	Monthly Starting 8 days after arriving (11 weeks old) and until 28 weeks old	Specific antibodies Log transformed - LMM
Detection of *L. intracellularis* – qPCR from feces	Pen level *N* = 20 pens Pool of feces samples were sent to lab at the end of the study	Monthly Starting 8 days after arriving (11 weeks old) and until 28 weeks old	Number of positive pens over the total of pens
Selected biomarkers in saliva and blood	Individual level *N* = 40 pigs Saliva and blood samples were sent to the lab at the end of the study. Analyzed once the period of infection was determined through serology and qPCR.	Monthly Starting 8 days after arriving (11 weeks old) and until 20 weeks old	Haptoglobin (Hp) C-reactive protein (CRP) Total esterase activity (TEA) Adenosine deaminase (ADA) Normal - LMM
Body weight	Individual level *N* = 516 pigs All observers were involved to move the pigs	At entry (10 weeks old) and at 29 weeks old	Average daily gain (ADG) Normal - LMM

GLMM: Generalized Linear Mixed Models; LMM: Linear Mixed Models

### Monitoring of *Lawsonia intracellularis*

*L. intracellularis* had been previously detected in other batches at the facility. Fecal bacterial load was monitored to identify the timing and approximate magnitude of infection rather than to serve as a primary outcome. Measurements were obtained at pen level on a relatively small sample size (10 pens per treatment group) and at a limited number of discrete time points (11, 15, 20, 24, and 28 weeks of age). Pooled fresh fecal samples were stored cooled until analysis. Bacterial load was measured by qPCR (Bactoreal^®^ Kit *Lawsonia intracellularis*, Ingenetix GmbH, Vienna, Austria). Subclinical ileitis was defined by the absence of ileitis-associated clinical signs and/or mortality, as well as by the detection of the bacterium by qPCR [9, 29]. A low bacterial fecal load was considered when the quantification of genomic copies by RT-PCR (Bactoreal^®^, Kit *Lawsonia intracellularis*, Ingenetix GmbH, Vienna, Austria) [30] did not exceed 4 Log10 GE copies/µL.

To estimate the timing of the specific antibody response, longitudinal serum samples were obtained from two pigs per pen (84 pigs in total), randomly selected within each pen based on RFID identification following allocation to the finishing unit. These pigs were individually monitored throughout the study through repeated blood and saliva sampling. Blood collection was performed on the exact same dates as fecal sampling. Samples were centrifuged and stored at -20 °C until analysis. Serum antibodies were analyzed using a commercial blocking ELISA (SVANOVIR^®^ L. intracellularis/Ileitis-Ab, INDICAL BIOSCIENCE, Sweden) following the manufacturer’s instructions. Antibody levels were expressed as percent inhibition (PI) of the negative control, where PI = 100 - (ODsample / ODnegative control) x 100, and OD represents the optical density measured by the ELISA reader.

Fecal consistency was assessed by visual inspection using a four-point ordinal scale (0 normal; 1 mild diarrhea; 2 moderate diarrhea; 3 severe diarrhea, watery feces and/or with incorporation of blood) to identify signs consistent with diarrhea at pen level [31].

### Behavioral observations through video recordings

Weekly assessments of pig behavior were performed from 11 weeks of age (7 days after entry at the finishing unit) until 28 weeks of age (one week before the end of the finishing period) during daytime by video recording from the 42 pens included in the study. Video cameras (dome Gamma 1080p ULTRA Sony© 2.13 Mpx Exmor IMX307) were installed, and recordings were stored in four video recorders. Observations were recorded at pen level. Behavioral observations included tail-directed behaviors as the primary outcome. In addition, active behaviors (e.g., exploration) and postures were recorded as additional behavioral measures. Both scan sampling and focal sampling were used. Each one of these observational methods was done by a single observer to avoid inter-observer bias. Observers were blinded to the treatment.

#### Focal sampling

Video recordings were analyzed by observer number 1 for the first 5 min of each hour from 8:30 to 11:30 (4 repetitions of 5 min each in the morning) and from 18:30 to 19:30 (2 repetitions of 5 min each in the evening) using focal sampling. This resulted in 30 min observation per day per pen and a total of 378 h of observation across the entire finishing period. The ethogram is presented in Table 2.

**Table 2:** Ethogram used to register pig’s behavior (recorded by video-camera) by focal sampling

Oral manipulation	Description
Nosing tail region/rear-end of the pig, referred to as “nosing tail region”	Touching, sniffing, rooting, chewing or biting the ano-genital region or immediate surroundings. It is counted as a new observation if the performer takes its head away from the receiver for more than 5 s and starts exploring the tail/anal region again.
Tail-in-mouth (TIM) and tail biting, referred to as “tail manipulation”	Chewing, sucking or biting a pen-mate’s tail. Includes clear manipulation of the tail, tail in mouth, mild, moderate, and severe tail biting behavior. It is counted as a new observation if the performer lets go of the tail and grabs it again, even immediately.
Belly nosing	Repetitive up and down snout movement on the belly of a pen mate. It is counted as a new observation if the performer takes its head away from the receiver for more than 5 s and starts belly nosing again.
Ear biting/Ear sucking	Taking the ear of a pen mate into the mouth or nibbling, sucking or chewing the ear. It is counted as a new observation if the performer lets go of the ear and starts sucking or biting again, even immediately.

(adapted from [32, 33])

#### Scan sampling

Once every 30 min, pigs’ behavior was recorded by observer 2 using instantaneous scan sampling during the periods of high activity from 8:30 to 11:30 and from 18:30 to 19:30, resulting in 12 scans per day per pen. The ethogram is presented in Table 3.

**Table 3:** Ethogram used to register pig’s behavior (recorded by video-camera) by instantaneous scan sampling

General activity	Description
Nose/exploring enrichment	Touching, sniffing, rooting or biting the enrichment
Exploring pen features	Touching, sniffing, rooting or licking the floor or pen walls
Feeding/Drinking	Head in feeder and/or chewing food in proximity of feeder, with head directed towards feeder. Head in drinker/snout in contact with nipple.
Penmate-directed interaction	Sniffing, nosing, licking and moving gently away from the animal without an aggressive or flight reaction from this individual. Social negative interactions, including biting or any social behavior with a fearful or aggressive response from the disturbed animal.
Other active behavior	All other active behaviors not included in any of the above categories.
Non-active behavior	Resting in a lateral or sternal position, including pigs that were resting with eyes opened

**Posture**	**Description**
Standing motionless	Standing upright on 4 legs in one location without moving legs directionally, possible movements of head/body parts and minor movements of legs
Standing walking	Activity of all 4 legs resulting in directional movement/change in location.
Lying ventrally	Body weight is supported by belly and sternum in contact with the floor. The animal is lying on its sternum, with shoulder not touching the floor.
Lying laterally	Body weight supported by side with the shoulder in contact with the floor. The animal is lying on its side, with one shoulder having contact with the floor
Sitting	Body is supported by hindquarter, and the front legs are straight.

(adapted from [15, 34])

### Tail lesion scoring – at the farm

Tail lesions were assessed individually at arrival at the finishing building (10 weeks old) and once a week, every 7 days, until the end of the finishing period. Tail lesions were scored from inside the pen by one trained observer (Observer 3) who remained consistent throughout the study. At first, pigs were called from outside the pen and approached the evaluator voluntarily. The evaluator then entered the pen, identified each pig via RFID ear tag, and manually inspected the tail by gently holding it to allow full visualization. When necessary, pigs were briefly distracted (e.g., with a small amount of straw) to facilitate accurate assessment. Tails contaminated with fresh liquid manure were cleaned with a cloth prior to evaluation. In cases of more adherent dirt, complete cleaning was not always possible, which could limit the detection of minor lesions. Therefore, minor superficial scratches and minor scabs were not classified as tail lesions in this study. All 516 pigs from the 42 study pens were assessed for tail lesions using the scoring system presented in Table 4. This scale has been adapted from [34] and [35], combining tail damage and wound freshness scoring. To ensure intra-observer repeatability with multi-colored pig tails (white, brown, black) and tails dirty with feces, minor scratches and minor scabs on the tail were not considered. Healed, shortened tails were classified as “no clear tail lesion” (category 0), as the assessment focused on active or recent tail lesions.

**Table 4:** Tail lesion scoring system

Tail lesion scoring	Description
0	No clear tail lesion. This includes minor superficial scratches or minor scabs on the tail. It also includes healed, shortened tails.
1	Visible wound which includes clear hard dry scab, scab with tissue damage, the scab is not intact showing some tissue underneath and older blood
2	Fresh visible red wound, dry red blood, not bleeding
3	Fresh visible red wound, bleeding

Daily observations of severe tail damage were controlled by the stockperson. An aluminium-based spray (Aluspray^®^) was applied to tails with visible fresh wounds as soon as these were detected. This product forms a protective barrier over the wound, helping to reduce contamination, limit exposure to flies, and supports healing. It may also help to reduce bleeding and decrease the attractiveness of fresh wounds to other pigs. In addition to the spray treatment, supplementary enrichment materials were provided in the pen (e.g., Easy Fix^®^ luna). When recurrent or repeated bleeding from the tail was observed, the affected pig was separated overnight for recovery and reintroduced to its original pen in the morning. If two or more pigs in a pen presented fresh wounds with bleeding on more than two consecutive days, the affected pen was opened and all pigs were given access to a « play pen » equipped with toys and rubbing walls for 12 h, after which they were returned to their original pen. These measures allowed full control of tail biting outbreaks at pen level.

### Determination of adenosine deaminase, acute phase proteins and total esterase activity concentrations

Of the 20 pens selected for qPCR of fecal samples and of the 84 randomly selected pigs used for serological detection of specific antibodies, a subset of 40 pigs (two pigs per pen) was chosen for the determination of a panel of biomarkers including haptoglobin (Hp), C-reactive protein (CRP), total esterase activity (TEA), and adenosine deaminase (ADA). Repeated measurements were taken monthly on the same selected pigs. Saliva and blood samples were collected on the same day, with saliva sampling performed prior to blood collection. Saliva was obtained by allowing each pig to actively grab and chew a Salivette^®^ attached to a metal rod for at least 30 s. The sponges were then placed into plastic tubes and centrifuged at 3,000 × g for 10 min at 4 °C. The recovered saliva was aliquoted and stored at − 80 °C until analysis of ADA and TEA. ADA was analyzed with a commercially available spectrophotometric assay (Adenosine Deaminase assay kit, Diazyme Laboratories, Poway, CA, USA), previously validated for porcine saliva [27]. Salivary TEA was measured by a method based on the hydrolysis of the substrate 4-NA (Sigma-Aldrich Co, St Louis, Mo, USA) by salivary esterases, as previously reported in pigs [25]. Blood samples for CRP and Hp determination were collected from the jugular vein, centrifuged at 2,000 × g for 5 min, and the resulting serum was stored at − 80 °C until further analysis. Porcine C-Reactive Protein (CRP) and Haptoglobin (Hp) concentrations were measured using an automated biochemistry analyzer (Olympus AU2700, Olympus Diagnostica GmbH, Hamburg, Germany) previously validated in pigs [36, 37].

### Performance and mortality

Body weight was measured individually with MS Pigscale for finishing pigs (MS Schippers^®^), synchronized with LeeO system, at entry (10 weeks old) and prior to slaughter (29 weeks old). Mortality during the growing-finishing period was recorded by the farmer as an additional outcome in the present study.

### Statistical analysis

The pen was the statistical unit for all behavioral data (42 pens) and tail lesions data (42 pens). Behavioral recordings from scan sampling were presented as the prevalence of observation periods in which the behavior in question was performed. The percentage of active pigs was expressed as a proportion of the total number of pigs observed. Categories of active behaviors were expressed as a proportion of the number of active pigs. Behavioral recordings from focal sampling were presented as the number of events per 10 pigs within 30 min. Tail lesions were expressed as the number of affected pigs in each tail lesion category relative to the total number of pigs assessed per pen. The pig was the statistical unit for specific antibodies (84 pigs), inflammatory biomarkers (40 pigs), average daily gain (ADG), and mortality (number of deaths relative to the total number of pigs). ADG and mortality were calculated for each treatment group, considering all animals present in the barn (516 pigs). ADG was calculated by weighing pigs on the day of transferring to the finishing unit and the day of transferring to the slaughterhouse and dividing by the number of days spent at the finishing unit. The specific antibody variable was log-transformed.

Continuous (antibodies, biomarkers, ADG), count (behavioral variables from focal sampling), and binomial response variables (behavioral variables from scan sampling, tail lesions) were analyzed using the appropriate generalized linear mixed model. All models included pen or animal as random factors depending on the availability of individual or group measurements and considered subject-specific random intercepts and slopes. These models also included treatment group, sex, age, and their interactions as fixed effects.

All models were validated by examining the residual plots, goodness-of-fit-indices and presence of overdispersion in count data.

Statistical decisions were made based on a significance level of 0.05. No corrections for multiplicity of contrasts were applied.

Data management and analysis were performed using SAS v9.4 (SAS System, SAS Institute Inc., NC, USA, 2025).

## Results

Baseline characteristics and performance of vaccinated and unvaccinated pigs are presented in Table 5. To enable a biologically meaningful interpretation of behavioral outcomes and tail lesion data, the timing of *Lawsonia intracellularis* infection is presented first, followed by the respective outcome measures.

**Table 5:** Baseline characteristics and performance of vaccinated and unvaccinated pigs during the study period

	Vaccinated	Unvaccinated	*p*-value
Number of pens	42	42	-
Number of pigs	258	258	-
Females, n (%)	137 (53%)	137 (53%)	
Males, n (%)	121 (47%)	121 (47%)	-
Weight at entry, 10 weeks of age (kg, mean ± SD)	22.9 ± 4.3	23.1 ± 4.9	0.69
Weight at 29 weeks of age (kg, mean ± SD)	132.7 ± 14.6	132.6 ± 15.8	0.76
ADG (g/day, mean ± SD)	832 ± 98	830 ± 96	0.94
Mortality, n	20	20	-

### Monitoring of *Lawsonia intracellularis*

qPCR (Table 6) and serological data (Fig. 2) were used to monitor exposure and to define the timing of infection rather than as a primary study outcome.

All faecal samples were qPCR-negative for *L. intracellularis* at the beginning of the monitoring period (11 weeks of age). Shedding was detected at 15 weeks of age in two pens from each group, with low bacterial loads ranging from 0.5 to 3.0 log_10_ copies/µL. Of the five sampling time points, the highest bacterial load was detected at 20 weeks of age, at which point the proportion of positive pens reached 80% (8/10) in the unvaccinated control group and 50% (5/10) in the vaccinated group (Table 6). At 24 weeks of age, all animals had cleared the infection and remained negative until the end of the study (28 weeks of age).

No evidence of clinical signs compatible with severe diarrhea was detected in either the vaccinated or the unvaccinated groups during the study.

**Table 6:** Detection of *L. intracellularis* through qPCR of feces at pen level (*N* = 20 pens) expressed in number of positive pens over the total of pens assessed at different time points

	11 weeks old	15 weeks old	20 weeks old	24 weeks old	28 weeks old
Unvaccinated (*N* = 10 pens)	0/10	2/10	8/10 Median of bacteria load 1.4 log_10_ copies/ µL	0/10	0/10
Vaccinated (*N* = 10 pens)	0/10	2/10	5/10 Median of bacteria load 0.2 log_10_ copies/ µL	0/10	0/10

Specific antibodies against *L. intracellularis* are presented in Fig. 2. Vaccinated pigs showed higher ELISA PI values at all time points except at 20 weeks of age. In both groups, a marked increase in ELISA PI values was observed between 15 and 20 weeks of age, indicating seroconversion during this interval, after which antibody concentrations remained elevated.

### Behavioral observations

**Fig. 3:**
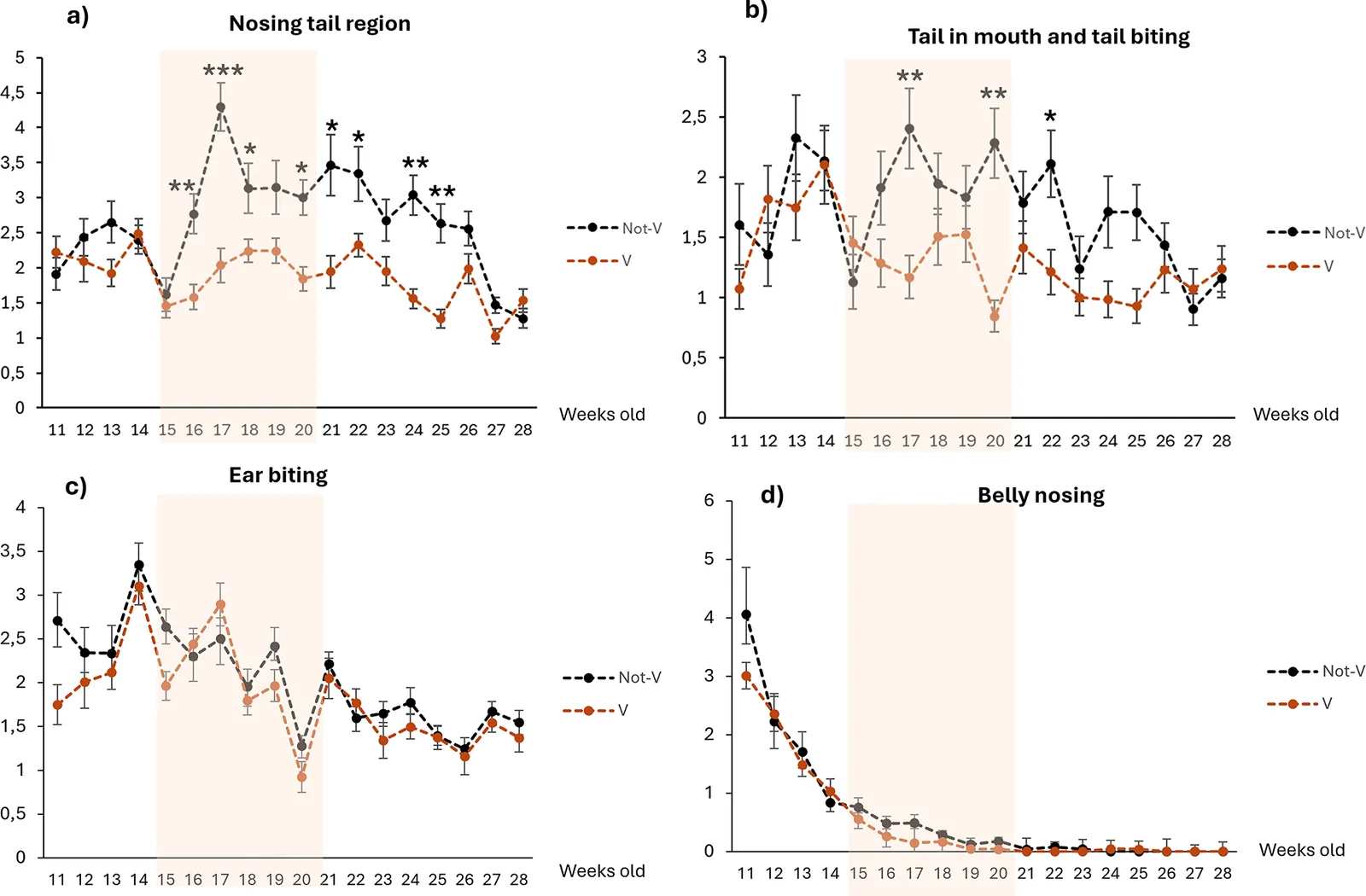
Nosing tail region (**a**), tail manipulation (**b**), ear biting (**c**) and belly nosing (**d**) assessed by focal sampling for each finishing week (expressed in weeks old) and expressed as events (Mean+-SE) per 10 pigs for 30 min observation. Significant differences between the two treatment groups (unvaccinated = Not-V and vaccinated = V) are shown. * *p* < 0.05, ** *p* < 0.01, *** *p* < 0.001. The period of *L. intracellularis* detection in feces is shown with an orange box

Focal observation results are shown in Fig. 3. Nosing of the tail region/rear-end (referred to as “nosing tail region”) was significantly higher in unvaccinated pigs between 16 weeks of age and 25 weeks of age. Tail manipulation was significantly higher in unvaccinated pigs at 17, 20, and 22 weeks of age. Belly nosing and ear biting/sucking were similar between treatment groups. Overall, females (2.7 ± 1.9 events/10 pigs/30 min) exhibited more nosing of the tail region than males (1.7 ± 1.3 events/10 pigs/30 min; *p* < 0.001).

**Fig. 4:**
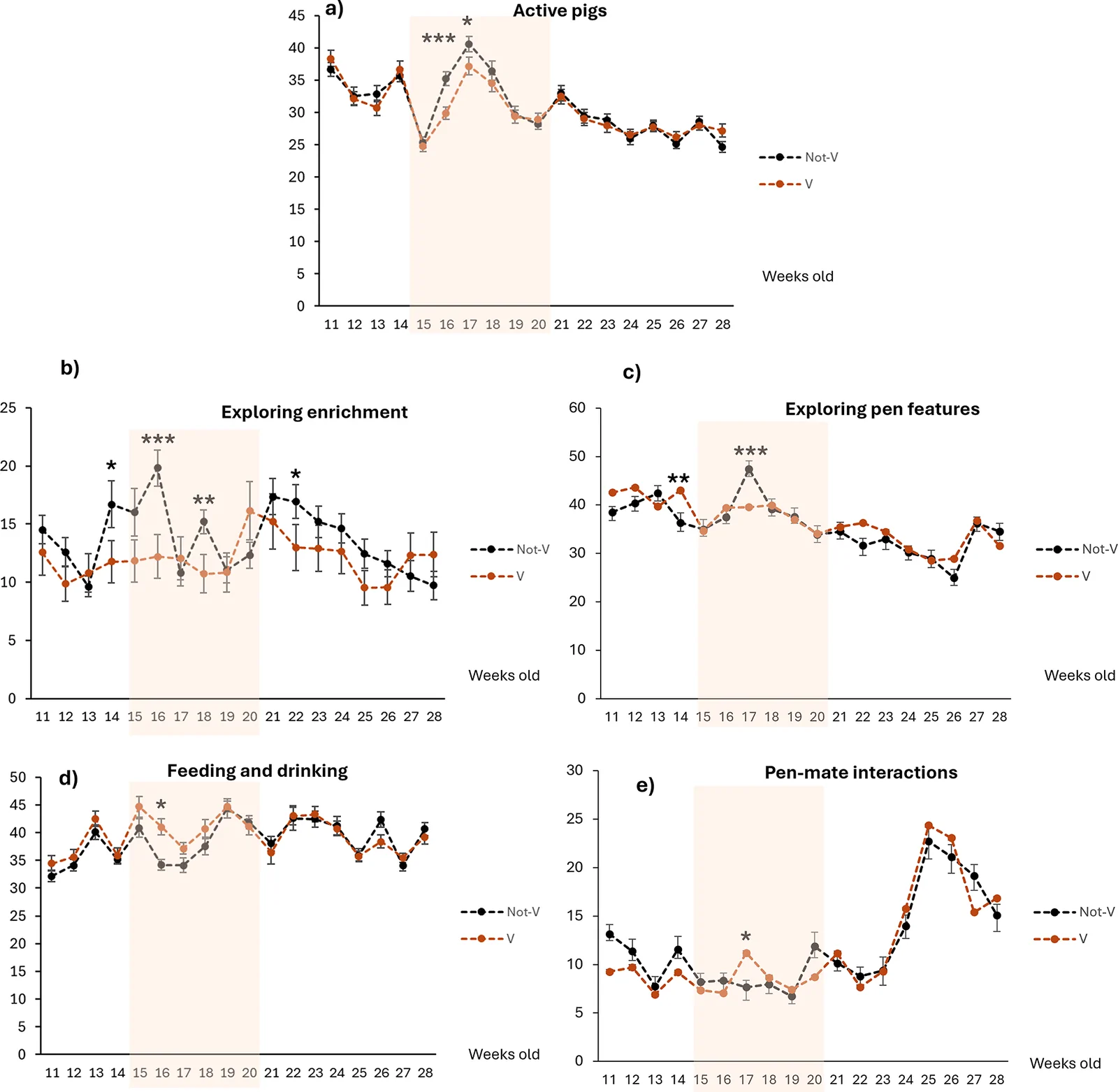
Percentage (mean and SE) of active pigs (**a**), exploration of the material (**b**), exploration of the pen (**c**), drinking-feeding behaviors (**d**), pen mate interactions (**e**) assessed by scan sampling for each finishing week expressed in weeks of age (age of the pigs). Significant differences between the two treatment groups (unvaccinated = Not-V and vaccinated = V) are shown. * *p* < 0.05, ** *p* < 0.01, *** *p* < 0.001. The period of *L. intracellularis* detection is shown with an orange box

Across all observation periods, 35% of pigs were active throughout the finishing period during daytime. The percentage of active pigs was similar between treatment groups except at 16 weeks of age and 17 weeks of age where unvaccinated pigs were more active (week 16: OR = 1.3, 95% CI [1.1–1.5]; week 17: OR = 1.1, 95% CI [1.0–1.3]) (Fig. 4a) with increased exploration of the pen observed at 17 weeks of age (OR = 1.4, 95% CI [1.1–1.6]) (Fig. 4c). Unvaccinated pigs manipulated enrichment materials significantly more at 14, 16, 18 and 22 weeks of age (week 14: OR = 1.4, 95% CI [1.1–1.8]; week 16: OR = 1.3, 95% CI [1.3–2.3]; week 18: OR = 1.4, 95% CI [1.1–1.9]; week 22: OR = 1.4, 95% CI [1.1–1.8]) (Fig. 4b). Conversely and more sporadically, unvaccinated pigs showed fewer pen mate interactions at 17 weeks of age (OR = 0.6, 95% CI [0.5–0.9]) (Fig. 4e) and less drinking/feeding behavior at 16 weeks of age (OR = 0.8, 95% CI [0.6–0.9]) (Fig. 4d).

Postures showed very limited differences between groups over time. During the period of *Lawsonia intracellularis* detection (15–20 weeks of age), unvaccinated pigs displayed a higher proportion of “standing walking” at a specific time point (week 17: unvaccinated 24% ± 8.5% vs. vaccinated 19% ± 6.6%; OR = 1.3, 95% CI [1.1–1.6]). In contrast, standing motionless remained similar between groups throughout the study (12% vs. 12%). Likewise, lying laterally (unvaccinated 47% vs. vaccinated 48%) and sitting (unvaccinated 3.4% vs. vaccinated 3.5%) did not differ between treatments at any time point. Overall, postural measures were largely comparable between groups, with only a transient increase in standing and walking observed in unvaccinated pigs.

### Tail lesions

**Fig. 5:**
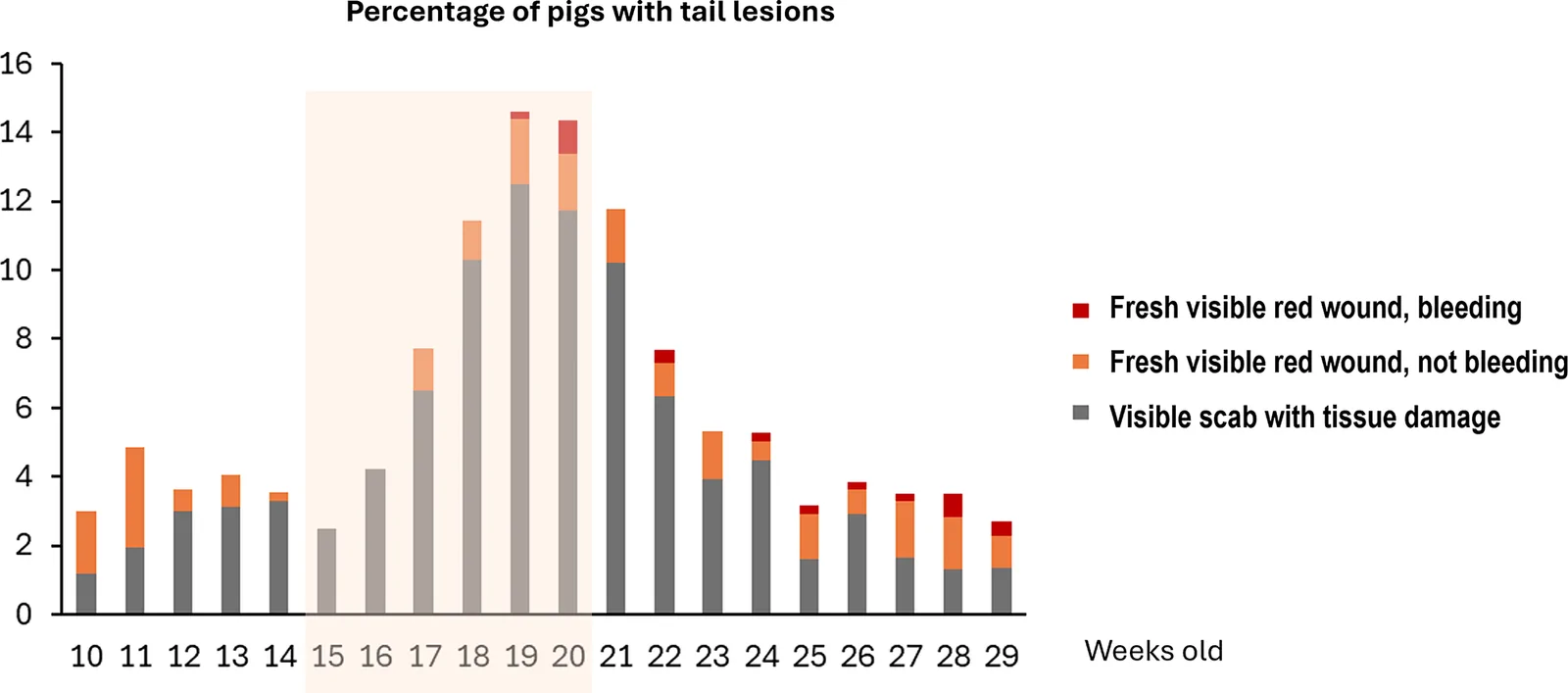
Percentage of pigs with different tail lesion scores throughout the finishing period. The period of *L. intracellularis* detection is shown with an orange box

**Fig. 6:**
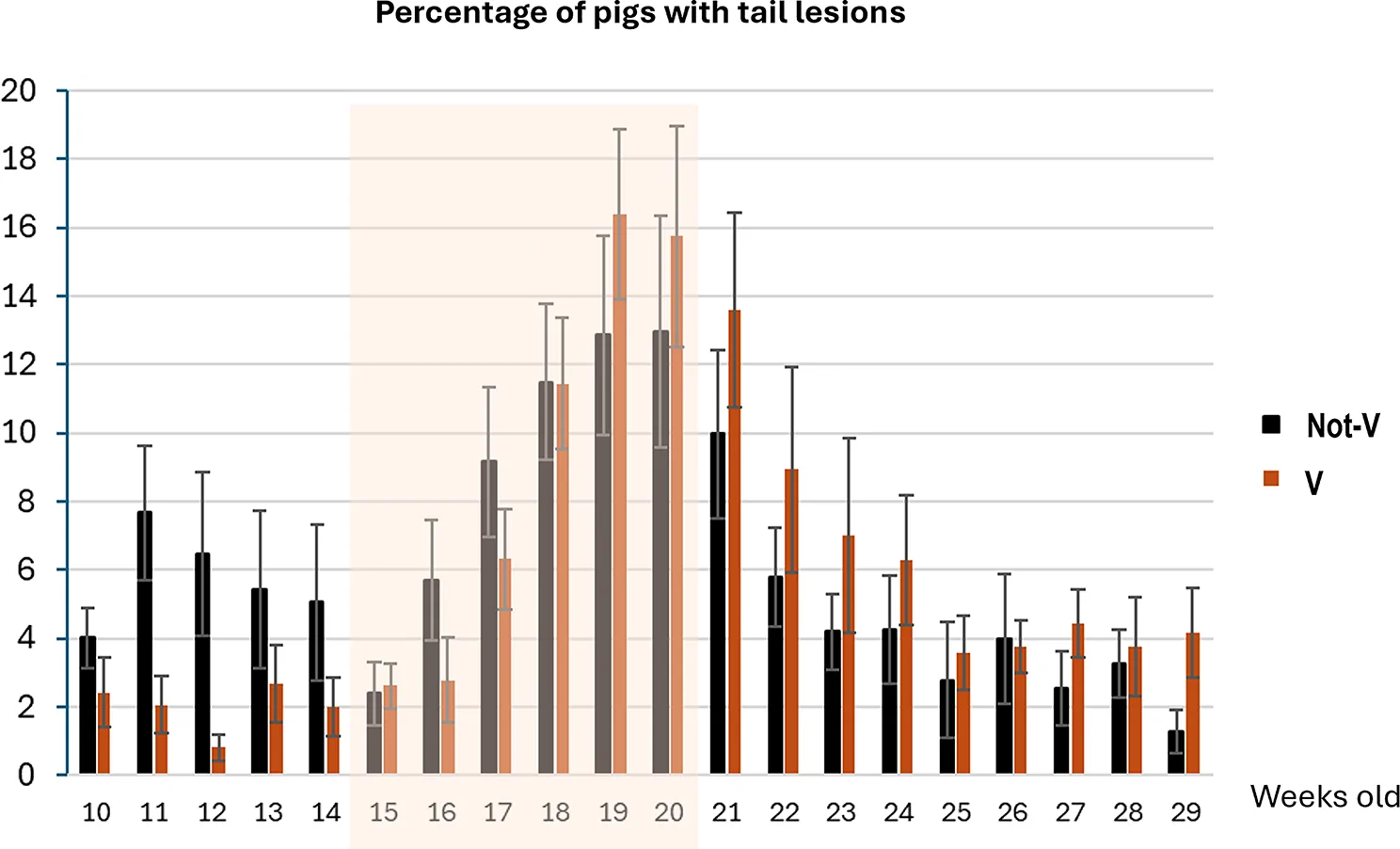
Percentage of pigs with tail lesions (sum of all scores of tail lesions) across the finishing period and depending on the treatment group. No significant differences between treatment groups were observed at any time point. The period of *L. intracellularis* detection is shown with an orange box

Distribution of tail lesions across the finishing period is shown in Fig. 5. For the entire growing-finishing period, average prevalence of tail lesions was 4.2% (scab with older blood), 1% (fresh wound not bleeding) and 0.1% (fresh wound bleeding). The highest prevalence of tail lesions appeared between 18 weeks of age and 21 weeks of age reaching a maximum value of 14.6% (± SD 17.4%) of pigs affected at 19 weeks of age (Fig. 5). Prevalence of tail lesions did not vary significantly depending on the treatment group (Fig. 6).

### Adenosine deaminase, acute phase proteins and total esterase activity

**Fig. 7:**
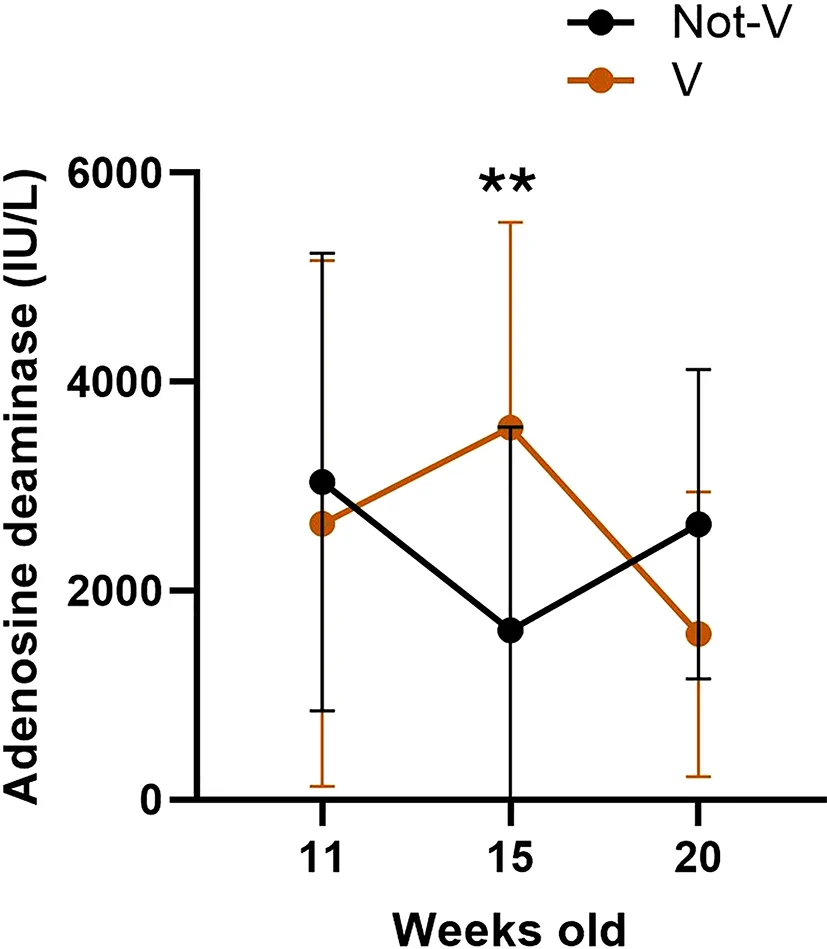
Adenosine deaminase (ADA) concentrations (mean and SD) at different time points in unvaccinated (black) and vaccinated (orange) pigs. Significant differences between the two treatment groups (unvaccinated = Not-V and vaccinated = V) are shown. * *p* < 0.05, ** *p* < 0.01, *** *p* < 0.001

ADA activity was higher in vaccinated pigs at 15 weeks of age (*P* = 0.007), while no differences were observed at other time points (Fig. 7). Haptoglobin, C-reactive protein and esterase activity concentrations did not differ between groups at any time point (Table 7).

**Table 7:** Haptoglobin (Hp), C-reactive protein (CRP), total esterase activity (TEA) concentrations (mean and SD) at different time points in unvaccinated (Not-V) and vaccinated (V) pigs

		11 weeks old	15 weeks old	20 weeks old
Hp (mg/L)	Not-V	1.8 ± 0.6	1.3 ± 0.6	0.7 ± 0.6
	V	1.6 ± 0.6	1.0 ± 0.6	0.7 ± 0.5
CRP (µg/mL)	Not-V	33.6 ± 25.2	20.2 ± 24.3	10.9 ± 9.2
	V	32.6 ± 24.6	15.5 ± 16.5	12.7 ± 13.6
TEA (IU/L)	Not-V	275.3 ± 306.0	536.6 ± 455.1	337.6 ± 412.4
	V	287.0 ± 2517.4	595.0 ± 823.1	338.9 ± 299.9

### ADG and mortality

Average daily gain (ADG) and mortality were similar between treatment groups (Table 5). In the vaccinated group, 11 losses were attributed to respiratory disorders, 3 to severe anemia, and 6 pigs that appeared clinically healthy were found dead. In the unvaccinated group, 11 losses were attributed to respiratory disorders, 1 to severe anemia, 2 to meningitis, and 6 pigs that appeared clinically healthy were found dead without a definitive cause.

## Discussion

Pigs vaccinated against *Lawsonia intracellularis* displayed a lower occurrence of nosing of the tail region and tail manipulation (including tail-in-mouth, TIM). However, tail lesion prevalence did not differ between treatment groups. These behavioral reductions were most evident during the period of *L. intracellularis* excretion, suggesting that vaccination may influence early stages of tail-directed behavior, potentially by modulating health status and associated behavioral responses. Nosing the tail region and tail-in-mouth (TIM) are considered early manifestations of tail biting, which may precede damaging tail biting but do not necessarily result in lesions [33, 38, 39]. Although TIM does not necessarily result in immediate lesions, it reflects increased oral manipulation directed towards pen mates and may indicate underlying behavioral or physiological drivers such as discomfort, stress, or exploratory motivation. The observed reduction in nosing of the tail region and tail manipulation therefore suggests that vaccination may influence early stages of tail biting behavior.

The more pronounced increase in nosing of the tail region and TIM in unvaccinated pigs suggest a link between gastrointestinal status and behavioral expression. On the performer side, pigs experiencing suboptimal health may show increased exploratory and redirected oral behavior, particularly towards the tail region [40, 41]. Infection with *L. intracellularis* is known to alter gut microbiota and intestinal function [42, 43], and associations between tail biting and fecal short-chain fatty acid (SCFA) profiles [44] suggest a potential role of microbial metabolites in modulating behavior via the gut–brain axis. On the receiver side, infection-related changes may also modify cues emitted by affected pigs, potentially increasing investigatory attention from pen mates towards the rear region. Given pigs’ highly developed olfactory abilities, they are likely to perceive subtle health-related odor differences in pen mates [33]. Although the underlying mechanisms remain unclear, such changes in odor or other chemical signals may contribute to the attractiveness of affected individuals and facilitate the initiation or escalation of tail-directed interactions. Notably, the same individual may act as both performer and receiver over time, reflecting the dynamic nature of these behaviors.

In addition to tail-directed behaviors, which were the primary outcomes, general active behaviors (exploration, feeding, and social interactions) and postures were also recorded as additional behavioral measures. During the period of *L. intracellularis* detection, unvaccinated control pigs interacted more intensively with enrichment materials at two out of six time points during *L. intracellularis* detection, as well as just before and after this period. Increased use of enrichment materials has been observed in pigs during early phases of behavioral unrest preceding tail biting outbreaks [40, 45]. In the present study, this may reflect increased exploratory motivation associated with subclinical gut discomfort. Differences in active behaviors were more sporadic: at certain time points within the period of infection, unvaccinated control pigs were more active and explored the pen more, while feeding/drinking and social interactions were slightly reduced. Minor postural differences were also observed with unvaccinated pigs spending more time walking at one time point. Such minor transient changes are coherent with fluctuating discomfort associated with subclinical diseases. Similar patterns have been reported in weaners under enteric or inflammatory challenges, including *Salmonella* infection or post-weaning diarrhea [46, 15]. These sporadic increases in activity support the hypothesis that gut discomfort may enhance exploratory and oral behaviors, particularly during early stages of infection in unvaccinated pigs. However, the more persistent increase in tail-biting related behaviors, especially nosing of the tail region, cannot be fully explained by changes in general activity, as these were sporadic and short-lived, suggesting that additional mechanisms may be involved. Pigs with higher *L. intracellularis* burden may attract increased investigatory attention from pen mates towards the perianal region, contributing to the development of tail-directed behaviors.

Despite increases in tail-biting related behaviors and exploration in unvaccinated pigs, tail lesion prevalence remained similar between groups. This apparent inconsistency is biologically and methodologically coherent. Tail lesions are the end-stage expression of a multifactorial process where not all tail biting events lead to a tail lesion [18, 19, 47]. Tail lesion scoring was based on direct visual inspection with manual handling of the tail; however, in some cases, soiling or limited visibility due to multicolored tails may have reduced the detection of minor lesions. Superficial scratches and small scabs were therefore not systematically recorded, which may have reduced sensitivity to subtle differences between groups. Furthermore, the welfare management protocol implemented to prevent escalation likely contributed to limiting lesion development. When early signs of tail lesions were detected, corrective interventions were promptly applied, including increased space allowance for several hours, application of aluminium-based spray to bleeding tails, and provision of supplementary enrichment material. While these interventions are consistent with good welfare practices, they may have reduced lesion incidence and severity across both treatment groups. This context emphasizes that tail lesion frequency should not be interpreted as a direct proxy of behavioral motivation when outbreak control measures are in place. It also points out that good management is a key measure to prevent tail biting damage.

Acute phase proteins, total esterase activity, and adenosine deaminase were assessed as complementary measures to behavioral outcomes. These variables provided physiological context and helped distinguish between systemic inflammation, stress- or pain-related responses, and immune activation. Acute phase proteins and total esterase activity did not differ between groups. This is consistent with the subclinical nature of the infection, where intestinal lesions may not have been sufficient to trigger a marked systemic inflammatory response. In pigs, acute phase proteins typically respond to severe or generalized inflammation, whereas mild or localized enteric disturbances often induce only limited systemic changes [22–24]. In addition, the short-lived kinetics of Hp and CRP may have obscured potential differences at the selected sampling time points [22]. TEA, a marker of autonomic activation and acute stress, also showed no differences between groups. This biomarker is more responsive to acute stressors such as pain or clinical conditions such lameness or prolapse [26] and may be less sensitive to low-grade or localized inflammation. These results suggest comparable levels of detectable systemic stress or pain between groups. In contrast, vaccinated pigs showed a transient increase in adenosine deaminase (ADA) activity at 15 weeks of age. ADA is involved in immune regulation and has been associated with T-cell activation and adaptive immune responses [48, 49]. In pigs, increased ADA levels have also been reported during inflammatory conditions such as gastrointestinal infections [50].

In the context of *Lawsonia intracellularis* infection, where protection relies primarily on cell-mediated immunity, this transient increase is consistent with vaccine-induced immune activation rather than disease-related inflammation [2, 51]. The absence of parallel changes in acute phase proteins supports the interpretation that ADA reflects a limited and transient immunological response rather than a generalized inflammatory state. Taken together the profile of these biomarkers suggests that vaccination primarily enhanced immune readiness (reflected by ADA) rather than suppressing a systemic inflammatory response (Hp, CRP) or acute stress linked to painful conditions (esterase). The reduction in tail-biting related behaviors in vaccinated pigs therefore appear to be linked to a better-controlled and localized enteric infection and a more balanced immune status, rather than to differences in overt systemic inflammation or physiological acute stress response.

No significant differences were observed between groups in mortality or average daily gain (ADG). This result aligns with previous studies showing variable production responses to *L. intracellularis* vaccination depending on disease pressure, management, and diet [2]. The lack of a performance difference reinforces that the primary benefit observed in this trial where *L.intracellularis* infection was subclinical with relatively low bacterial shedding was mainly behavioral rather than purely productive. Reductions in discomfort and tail-biting related behaviors have important welfare implications, even in the absence of measurable performance gains.

## Conclusions

Under natural subclinical *Lawsonia intracellularis* infection, vaccinated pigs demonstrated fewer tail-biting related behaviors and lower levels of active behaviors compared with unvaccinated controls. Although tail lesion rates were similar between groups, likely due to effective welfare management measures, the observed behavioral differences suggest that vaccination against *L. intracellularis* can enhance pig welfare even in the absence of clinical disease. Therefore, controlling subclinical enteric infections through vaccination may serve as an indirect yet practical complementary strategy to reduce tail-biting behavior on commercial pig farms.

## Supplementary Information

Below is the link to the electronic supplementary material.

Supplementary Material 1

## Data Availability

Data is property of MSD Animal Health; they can be available however under reasonable request, but some restrictions might apply.

## Declarations

### Use of AI tools

The authors used ChatGPT (OpenAI, San Francisco, USA) to support the editing of the Introduction and Discussion sections by improving language clarity. All content was critically reviewed, verified, and edited by the authors, who take full responsibility for the accuracy and integrity of the final manuscript.

## Abbreviations

APP: Acute phase protein
ADA: Adenosine deaminase
ADG: Average daily gain
CRP: C-reactive protein
GI: Gastrointestinal
GLMM: Generalized Linear Mixed Models
Hp: Haptoglobin
IgG: Immunoglobulin G
*L. intracellularis*: *Lawsonia intracellularis*
LMM: Linear Mixed Models
ODsample: Optical Density of the sample
PI: Percent inhibition
qPCR: Real-time quantitative polymerase chain reaction
RFID: Radio-frequency identification
SD: Standard deviation
SE: Standard error
TEA: Total esterase activity
TIM: Tail-in-mouth
Not-V: Not-Vaccinated
V: Vaccinated
