# Supplementary material for: Vaccination against Lawsonia intracellularis reduced tail-biting related behaviors in a commercial pig herd
Source: Porcine Health Manag. 2026 Jul 16;12:53. doi: 10.1186/s40813-026-00530-4 (PMC13673825; doi:10.1186/s40813-026-00530-4)
Supplement: Supplementary file 1 [file 40813_2026_530_MOESM1_ESM.docx]

AF1 Figure 1 Evolution of environmental temperature across the fattening period expressed per hour (mean ±SE).

AF1 Figure 2 Evolution of Relative Humidity across the fattening period expressed per hour (mean ±SE).
